# Molecular mechanisms and therapeutic applications of huaier in breast cancer treatment

**DOI:** 10.3389/fphar.2023.1269096

**Published:** 2024-01-12

**Authors:** Ke-fei Luo, Lin-xi Zhou, Zi-wei Wu, Yuan Tian, Jun Jiang, Ming-hao Wang

**Affiliations:** ^1^ Department of Breast and Thyroid Surgery, First Affiliated Hospital of The Army Medical University, Chongqing, China; ^2^ Department of Emergency Surgery, Linyi People’s Hospital, Linyi, China

**Keywords:** huaier, breast cancer, cell death, complementary alternative medicine, prognosis

## Abstract

Breast cancer is one of the most common female malignant tumors today and represents a serious health risk for women. Although the survival rate and quality of life of patients with breast cancer are improving with the continuous development of medical technology, metastasis, recurrence, and drug resistance of breast cancer remain a significant problem. Huaier, a traditional Chinese medicine (TCM) fungus, is a type of Sophora embolism fungus growing on old Sophora stems. The polysaccharides of *Trametes robiniophila Murr* (PS-T) are the main active ingredient of Huaier. There is increasing evidence that Huaier has great potential in breast cancer treatment, and its anti-cancer mechanism may be related to a variety of biological activities, such as the inhibition of cell proliferation, metastasis, tumor angiogenesis, the promotion of cancer cell death, and regulation of tumor-specific immunity. There is growing evidence that Huaier may be effective in the clinical treatment of breast cancer. This review systematically summarizes the basic and clinical studies on the use of Huaier in the treatment of breast cancer, providing useful information to guide the clinical application of Huaier and future clinical studies.

## 1 Introduction

According to WHO data, breast cancer has become the most common cancer diagnosed worldwide (Trapani et al., 2022). Breast cancer accounts for 7%–10% of the incidence of systemic malignant tumors, with a higher incidence in women between 40 and 60 years old, before and after menopause (Rossi et al., 2019). The development of breast cancer is influenced by genetic factors, and the age of onset has been decreasing in recent years (Trapani et al., 2022). Conventional treatment for breast cancer includes surgical resection, radiation therapy, chemotherapy, endocrine therapy, and targeted therapy (Waks and Winer, 2019). Although the prognosis for breast cancer is getting better with the continuous development of medical technology (Fridrichova and Zmetakova, 2019; Guo et al., 2023; Liao and Cao, 2023), recurrence and metastasis remain major challenges because microscopic metastatic lesions may still be present following treatment of the primary lesion and may grow over time, eventually leading to recurrence and metastasis. Furthermore, drug resistance commonly occurs because of the high variability and compensatory adaptation mechanisms of cancer cells, leading to treatment failure (Garcia-Martinez et al., 2021). It is essential to develop new therapeutic strategies and drugs to treat breast cancer.

There is an increasing recognition of therapeutic potential of extracts used in TCM and it is now generally accepted that TCM offers important advantages such as multi-targeting mechanisms, lower toxicity, and a wide range of pharmacological actions, that are important in preventing tumor occurrence, inhibiting tumor foci formation, and preventing recurrence and metastasis (Wang et al., 2021). TCM and chemotherapy in clinical studies can synergistically increase anti-tumor effects, reduce toxic side effects, reverse multidrug resistance, improve body immunity, reduce cancer recurrence and metastasis rates, and reduce patient suffering while prolonging survival time (Li et al., 2022). Artemisinin has been proved to have the ability of inducing tumor cells apoptosis and inhibit tumor cells proliferation and metastasis *in vivo* (Yao et al., 2018; Zou et al., 2019), enhance the efficacy of other treatment (Zhou et al., 2010; Li et al., 2021; Zeng et al., 2023), and has been widely used in a variety of tumors such as breast cancer, lung cancer, and liver cancer (Gao et al., 2022). Radix Codonopsis contains many compounds, such as saponins, polysaccharides, and amino acids, which can induce cancer cells apoptosis and inhibit angiogenesis (Wang et al., 2011), enhance immunity, and reduce the side effects of chemotherapy and radiotherapy, nowadays it is widely used in the treatment of malignant tumors, such as lymphoma and osteosarcoma (Bailly, 2021).

*Trametes robiniophila Murr* has a very long history of clinical therapeutic use as a TCM fungus. At present, its finished product, Huaier is mainly used clinically in the adjuvant treatment of various malignant tumors. The polysaccharides of *Trametes robiniophila Murr* (PS-T) are the main effective ingredient of Huaier, which is mainly composed of monosaccharides, and also contains small amounts of amino acids, organic acids, and various trace elements (Hu et al., 2019). Huaier has been shown to have considerable potential in treating breast cancer (Pan et al., 2019) and inhibiting its proliferation (Gao et al., 2017; Wang et al., 2019c). Wang et al. also found that Huaier can reduce breast cancer cell viability, inducing apoptosis through the H19-miR-675-5p-CBL pathway (Wang et al., 2017). Huaier polysaccharide can also regulate epithelial-mesenchymal transition (EMT) by inducing autophagy to suppress breast cancer cell invasion and metastasis (Tian et al., 2021), thereby improving breast cancer prognosis (Yao et al., 2020). Previously, we found that Huaier granules inhibited breast cancer disease progression and significantly improved DFS and OS in patients with advanced triple-negative breast cancer (TNBC) (Wang et al., 2019). Furthermore, Huaier is an effective immunomodulator in the treatment of breast cancer patients (Sun et al., 2013; Li et al., 2016; Tian et al., 2021; Li et al., 2022), effectively inhibiting tumor stem cells (Zhang et al., 2013; Wang et al., 2014) and tumor-induced angiogenesis (Littlepage et al., 2010; Li et al., 2015b; Li et al., 2016). In summary, Huaier has broad anticancer function, and no significant side effects have been proven (Li et al., 2015b; Yao et al., 2020).

This review systematically summarizes the basic and clinical studies on Huaier in the therapy of breast cancer, and comprehensively demonstrates the extensive and effective anti-tumor effect of Huaier, providing useful information to guide the clinical application of Huaier and future clinical studies.

## 2 Huaier inhibits proliferation and migration of breast cancer

### 2.1 Huaier inhibits breast cancer cell proliferation

**FIGURE 1:**
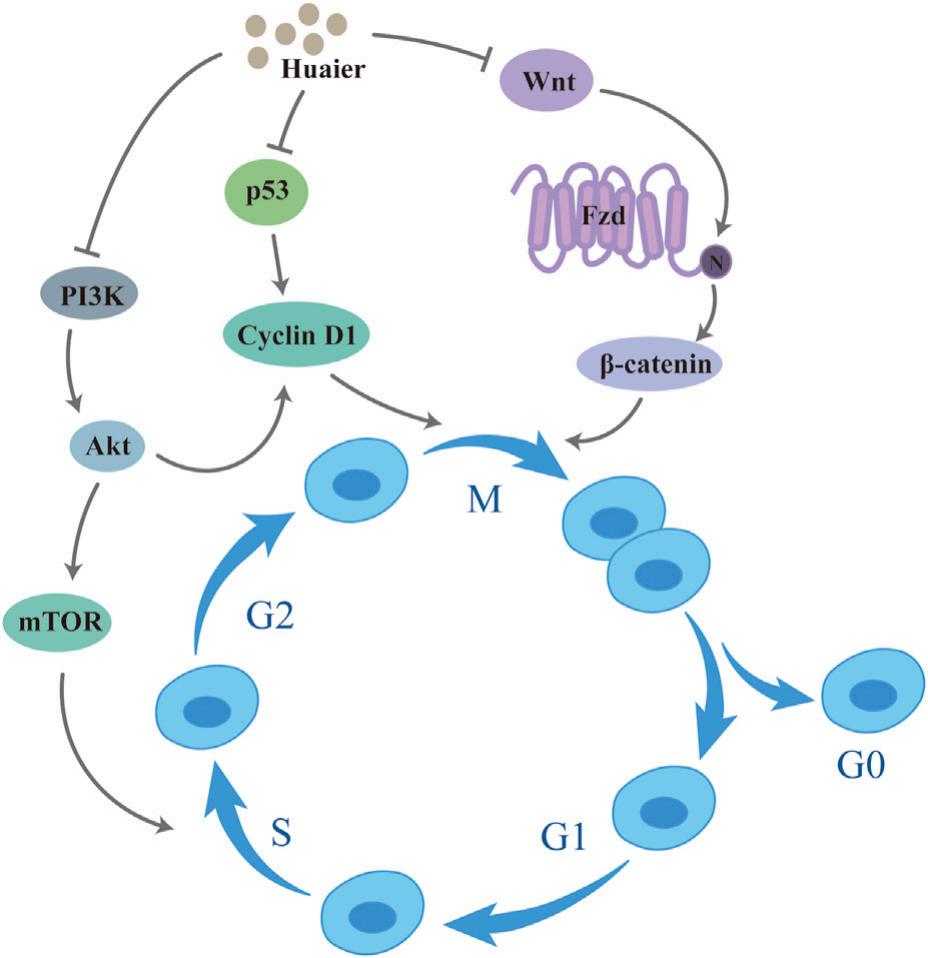
Huaier inhibits breast cancer cell proliferation.

The most important manifestation of tumor development is cancer cells proliferating uncontrollably (Hanahan, 2022). In an *in vivo* experiment, by plotting tumor growth curves, Wang et al. found that the growth of xenograft mammary tumors is inhibited in mice while using Huaier (Wang et al., 2015). Another study detected the nuclear antigen (Ki67) of proliferating cells in tumor tissues by immunohistochemistry and found fewer Ki67-positive cells and lower integrated optical density (IOD) of VEGF in BT474 tumor-bearing nude mice treated with Huaier compared with the control group, these *in vivo* experiments confirme that Huaier could inhibit the breast cancer cells proliferation (Liu, 2016). The experimental results of Qi et al. demonstrated that Huaier could enhance autophagy and block cell cycle by tamoxifen through Akt/mTOR signaling, and flow cytometry results showed that The combination of Huaier and tamoxifen resulted in a greater proportion of MCF-7 and T47D cells arrested in the G0/G1 phase and a smaller proportion in the S-phase. Analysis by protein blotting experiments revealed that cell cycle protein D1 expression, which is the major cell cycle protein in G1 phase, (Qi et al., 2016). Pan et al. used the CCK8 assay to observe the effect of different treatment times (24 h, 48 h) and different concentrations (2 mg/mL, 4 mg/mL, 8 mg/mL, 16 mg/mL) on the proliferation of TNBC cells, and found that Huaier inhibited the proliferation of breast cancer cells with a dose-time effect relationship (Pan, 2020). Experimental results of MTT assay and clone formation assay also showed that Huaier was effective in inhibiting the proliferation of breast cancer cells, and this inhibiting showed time dose dependence, inducing stagnation of cells at G0/G1 (Wang et al., 2012; Gao et al., 2017). It was also shown that Huaier induced cell cycle arrest in MCF-7 cells, but this effect was not shown on the MDA-MB-453 cell line (Zhang et al., 2010). Upregulation of p53 expression leads to cell cycle arrest, the study found that the expression of both p53 and phosphorylated-p53 (p-p53) increased in MCF-7 cells after Huaier treatment by protein blotting, suggesting that Huaier treatment promotes the accumulation and activation of p53 in MCF-7 cells (Zhang et al., 2010) (Figure 1).

### 2.2 Huaier inhibits breast cancer metastasis

Invasive metastasis of breast cancer is often considered the leading cause of patient death, and current studies have demonstrated that Huaier inhibits metastasis in the human breast cancer cell lines (Zhang et al., 2010; Song et al., 2015). This highlights the fact that Huaier has the therapeutic potential to be an effective anti-tumour metastatic treatment. Through MTT assay Zhang et al. found that the cell viability of MCF-7 and MDA-MB-231 cells decreased dramatically which are treated with 8 mg/mL Huaier. *In vitro* migration and scratch assays also demonstrated a decrease in breast cancer cell viability with Huaier treatment (Zhang et al., 2010). In the *in vitro* invasion assay, the number of invasive cells passing through the stromal gel-coated membrane was significantly lower in Huaier-treated MDA-MB-231 cells than in the control group, demonstrating that Huaier was effective in reducing the invasive potential of breast cancer cells (Zhang et al., 2010; Tian et al., 2021). Three types of cells, MDA-MB-231, MDA-MB-468, and MCF-7, also showed reduction in cell viability with Huaier (Wang et al., 2015).

**FIGURE 2:**
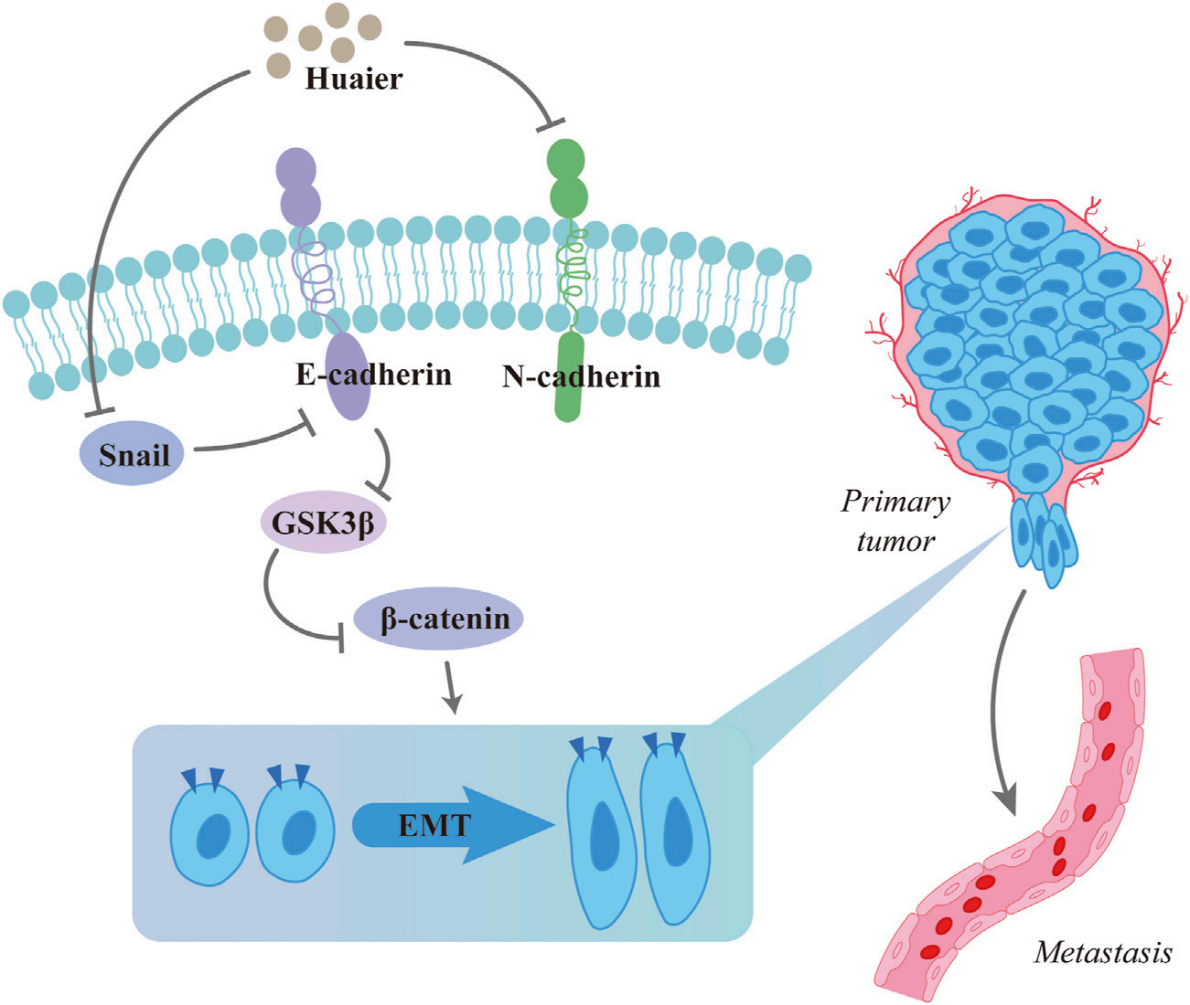
Huaier inhibits breast cancer metastasis.

Epithelial-mesenchymal transition (EMT) is the process which involves epithelial cells becoming cells with mesenchymal phenotypes and according to current studies, EMT plays a crucial role in cancer metastasis (Lin et al., 2021). A previous demonstration has been made, through transwell and cell scratch assays, that breast cancer cells are inhibited from migrating and invading by Huaier. EMT is initiated by snail, a key transcription factor, which inhibits E-cadherin/CDH1 expression and promotes cancer metastasis (Tran et al., 2011). Huaier specifically degrades Snail protein by inducing autophagy in an LC3-dependent manner, and prevents breast cancer cells from undergoing EMT. Moreover, in a mouse model of breast cancer lung metastasis, mice were treated with saline (controls) or 25 μg/g and 100 μg/g of Huaier by gavage on alternate days, for 21 days, and the results showed that in mice treated with Huaier, metastatic nodules decreased in size and number dose-dependently. Furthermore, mice treated with Huaier polysaccharide expressed more LC3 protein, while less Snail protein was expressed. This confirmed *in vivo* that Huaier polysaccharide induced the degradation of Snail by intracellular autophagy in breast cancer cells, thereby inhibiting EMT and decreasing lung metastatic nodule formation in mice (Tian et al., 2021) (Figure 2).

### 2.3 Huaier suppresses breast cancer stem cells

**FIGURE 3:**
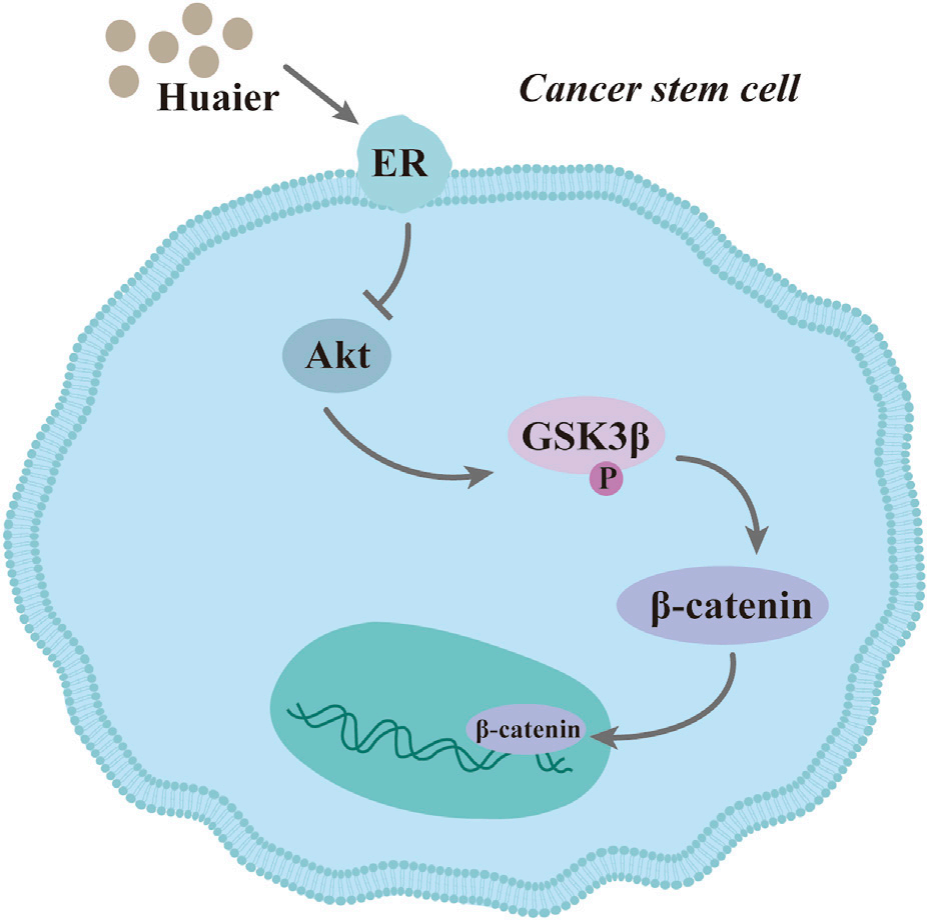
Huaier suppresses breast cancer sterm cells.

Cancer stem cells (CSCs) are one of the root causes of tumor recurrence (Gupta et al., 2009). Hu et al. showed that compared to that in the negative control group, the volume and number of breast cancer SUM-159 cell spheroid formation were significantly inhibited with increasing concentration of Huaier solution, considering that Huaier can effectively inhibit the stemness of SUM-159 (Hu et al., 2013). The results of a mammary gland sphere formation assay similarly indicated that triple-negative breast cancer stem cells are incapable of self-renewal in the presence of Huaier (Hu et al., 2019). ALDH1 is a common molecular marker of stem cells, and Huaier was found to significantly reduce the enrichment of ALDH1+ breast cancer cells depending on dosage. Huaier inhibited ERα-36-mediated AKT/GSK3β/β-catenin pathway to inactivate TNBC cells (Hu et al., 2013; Hu et al., 2019). The same finding was reported in the breast cancer MCF-7 cell line. And when MCF-7 cells were treated with Huaier for 24 h, the number of CD44+/CD24-cells was significantly decreased (Wang et al., 2014) (Figure 3).

### 2.4 Huaier inhibits angiogenesis within breast cancer

**FIGURE 4:**
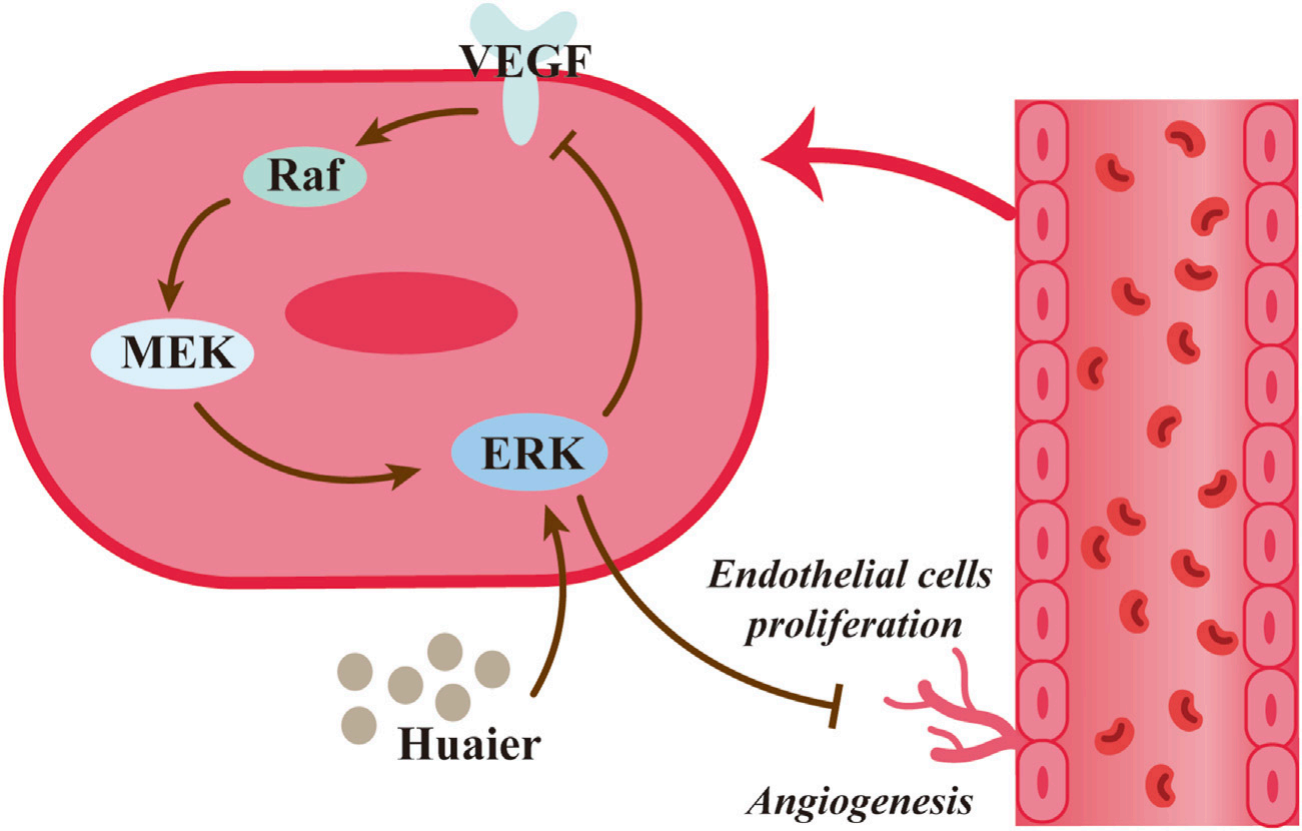
Huaier inhibits angiogenesis within breast cancer.

Angiogenesis is one of the typical features of tumor development, and as well as providing oxygen and nutrients to cancer cells, neovascularization promotes metabolic disorders, tumor dissemination, and metastasis (De Palma et al., 2017). Therefore, anti-angiogenesis is an important research direction in current anticancer therapy. Wang et al. showed that Huaier resulted in a marked dose-dependent reduction in aortic germination length and density. In addition, Huaier was found to cause rearrangement of the human umbilical vein endothelial cell line (HUVEC) skeleton, and Huaier also inhibited the proliferation of HUVEC using MTT assays. The results of modified scratch assay and cell migration assay showed a dose- and time-dependent inhibition of HUVEC migration ability with Huaier. The results of the current *in vitro* and *in vivo* experiments demonstrate the anti-angiogenic activity of Huaier (Wang et al., 2012). Vascular endothelial growth factor (VEGF) is a highly specific mitogen and potent vascular permeability enhancer of vascular endothelial cells. The proliferative, migration, and antiapoptotic functions of VEGF during angiogenesis (Musumeci et al., 2012). Studies have shown that Huaier inhibits the expression of VEGF by activating its upstream signal ERK and that this inhibition is dose-dependent (Wang et al., 2012; Zhang et al., 2020). Matrix metalloproteinase (MMP) is also a tumor perivascular infiltration-associated substance (Littlepage et al., 2010), and Huaier was found to decrease MMP concentrations in the serum of patients with hepatocellular carcinoma (Li et al., 2016), further demonstrating the anti-tumor angiogenic ability of Huaier (Figure 4).

## 3 Huaier promotes programmed cell death in breast cancer cells

**FIGURE 5:**
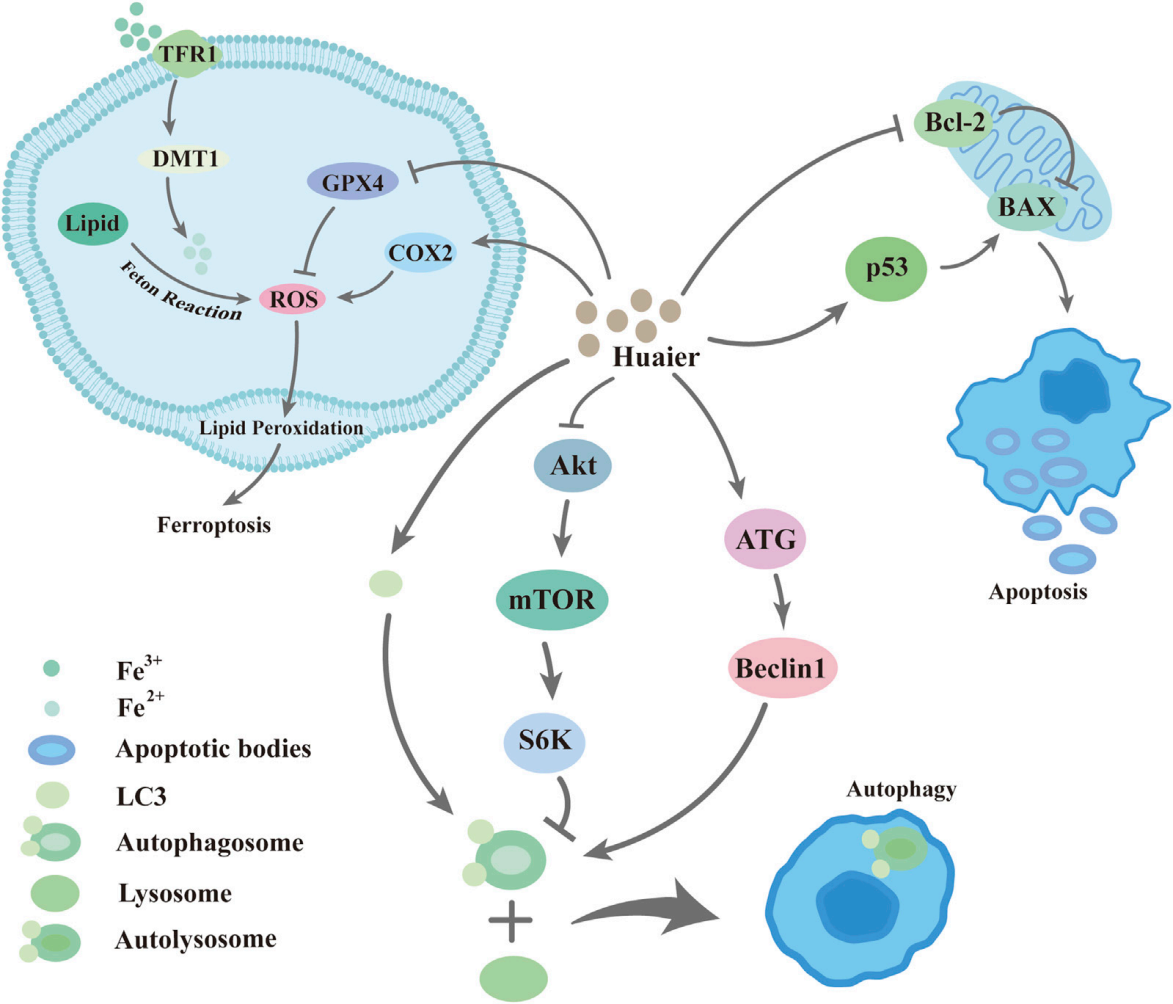
Huaier inhibits programmed cell death in breast cancer cells.

Programmed cell death (PCD) is a genetically determined active and ordered cell death. This process is a genetically regulated suicide protective measure initiated by cells when they encounter stimuli from internal or external environmental factors. It includes the induced activation of several molecular mechanisms and genetic programming by which non-essential cells or cells about to undergo specialization are removed from the body. PCD includes apoptosis, cellular autophagy, ferroptosis, necrotic apoptosis, cell scorching, and other modalities. Studies have shown that Huaier is effective in promoting programmed cell death in breast cancer cells (Luo et al., 2016; Qi et al., 2016; Liu et al., 2018) (Figure 5).

### 3.1 Huaier promotes apoptosis in breast cancer cells

Apoptosis is the active, physiological process of cell death under certain physiological or pathological conditions, controlled by intrinsic genetic mechanisms and under the regulation of genes. Changes in cell morphology can be observed during this process, including cell crumpling, nuclear condensation, apoptotic vesicle formation, cytoskeletal disintegration, and cytosolic vesicle formation (Wyllie et al., 1980). Compared to untreated cells, morphology of MCF-7 cells and MDA-MB-231 cells treated with Huaier was altered with cytoplasmic vacuolization, suggesting that Huaier causes damage to breast cancer cells (Zhang et al., 2010). Furthermore, after Huaier treatment, MCF-7 and MDA-MB-231 cells showed increased apoptosis rates and cell mortality (Zhang et al., 2010). Morphological changes also occurred in SUM-159 cells after Huaier treatment and some cell death was visible, and the IC50 of Huaier on SUM-159 cells could be calculated to be approximately 10 mg/mL by plotting the survival curve (Hu et al., 2013). Other studies have also shown a significant increase in the percentage of positive TUNEL staining in breast cancer cells treated with Huaier (Wang et al., 2012; Qi et al., 2016). Activation of p53 leads to apoptosis (Liu et al., 2023). Zhang et al. showed that p53 accumulated and was activated in MCF-7 cells in response to Huaier treatment (Zhang et al., 2010). In addition, Huaier induces cysteine activation, inhibits Bcl-2 expression, and upregulates BAX expression depending on time and dose. Therefore, Huaier can cause mitochondria-mediated apoptosis through the regulation of the Bcl-2/BAX/cysteine aspartase pathway (Billen et al., 2008).

### 3.2 Huaier promotes autophagic death of breast cancer cells

Autophagy is the catabolic process of capturing and degrading damaged proteins and organelles in lysosomes. Wang et al. detected that human breast cancer cells treated with Huaier showed mesenchymal vesicles with a large diameter in their cytoplasm, which characterizes the occurrence of autophagy in cells. Using projection electron microscopy for further evaluation, breast cancer cells exposed for 48 h to 4 mg/mL Huaier displayed autophagic vesicles containing extensive degraded membrane structures or cellular material (Wang et al., 2015). Other experimental studies reported a similar effect of Huaier on breast cancer cell morphology. MCF-7 cells treated with 7 mg/mL of Huaier were significantly larger, irregularly shaped, spiny and with cytoplasmic vacuolization changes, while MDA-MB-231 cells became elongated and showed a specific “drawn” morphology (Zhang et al., 2010). Moreover, compared to control cells, Huaier-treated breast cancer cells showed higher fluorescence density and more monodansyl cadaverine (MDC)-labeled particles, a specific marker of autophagic vesicles. This result suggests that Huaier extract increases the recruitment of MDC to autophagosomes (Wang et al., 2015). LC3 is the first mammalian protein with a specific association with the membrane of autophagosomes (Liang et al., 2006). In breast cancer cells treated for 48 h with 3 mg/mL Huaier, punctate LC3 fluorescence increased in number and intensity, indicating that autophagy occurred. Immunoblotting assays showed a significant increase in the expression of several autophagy-related genes and a decrease in the selective autophagy target p62/SQSTM1 in Huaier-treated breast cancer cells (Wang et al., 2015). At the same time, the Huaier-induced decrease in p-mTOR also the downstream targets of this protein are severely dephosphorylated as a consequence, suggesting that Huaier extract effectively inhibits mTOR/S6K signaling (Wang et al., 2015), triggering autophagy in cancer cells (Nicklin et al., 2009). Qi’s study similarly confirmed that it is possible for Huaier to induce autophagy in cells synergistically in estrogen receptor (ER)-positive breast cancer cells with tamoxifen (Qi et al., 2016).

### 3.3 Huaier promotes ferroptosis in breast cancer cells

Ferroptosis, an iron-dependent, programmed cell death mode that is distinct from apoptosis and autophagy, is dependent on iron-mediated oxidative damage, increased iron accumulation, free radical production, and increased fatty acid supply, and lipid peroxidation, which are key to the induction of ferroptosis. It is now generally accepted that intracellular ROS accumulation is an important cause of ferroptosis in cancer cells (Qiu et al., 2023). Huaier can induce intracellular ROS accumulation, and by inhibiting ferroptosis, Huaier significantly lessens its inhibitory effect on lung cancer cells (Tian et al., 2020). The current studies showed that Huaier can induce breast cancer cell death by promoting increased ROS production (Wang et al., 2022; Chen et al., 2023). In pancreatic ductal carcinoma, Huaier upregulated the ferroptosis-related proteins COX2, SLC7A11 and GPX4, and the upregulation were suppressed by autophagy inhibitor, suggesting that Huaier promotes ferroptosis by inducing autophagy. (Zhu et al., 2022). The results of KEGG pathway analysis similarly showed that it can induce ferroptosis in breast cancer cells by directly triggering ROS production through the downregulation of GPX4 (Li et al., 2020).

## 4 Huaier enhances anti-tumor immune response within breast cancer

### 4.1 Huaier regulates macrophages

Several studies have found that polysaccharides of TCM have strong anticancer activity by increasing the size of macrophages or restoring the normal morphology of macrophages, upregulating the proliferative capacity of macrophages, and enhancing the phagocytic function of macrophages to enhance the immune capacity of the body (Huang and Gao, 2023). In the case of breast cancer, the immune system is involved in its development, progression and even metastasis. Immune cells are the main body burdened with the immune function of the body, and peripheral blood of breast cancer patients often shows changes in immune cells (Wu et al., 2016; Wang and Du, 2021). In existing studies, Huaier has been shown to regulate the immune cells of the body, which can contribute to its anti-tumor effects (Li et al., 2022).

Macrophages are important components of innate immunity. There is a general consensus that macrophages are the main inflammatory component during tumor progression and that their functional phenotype is altered in response to various microenvironmental signals generated by tumor and stromal cells (Qian and Pollard, 2010). It is recognized that macrophages in the tumor microenvironment have two distinct phenotypes: classically activated (M1) macrophages and alternatively activated (M2) macrophages, M2 has been proven to possess anti-inflammatory, pro-angiogenic, and pro-tumor properties (Mantovani et al., 2002). Macrophages produce nitric oxide (NO) upon cytokine stimulation, and numerous studies have now shown that NO-induced synthesis is the main mechanism of action of activated macrophages in tumor killing (Zhang et al., 2005).

In the tumor microenvironment, most macrophages are of the M2 type. In this regard, they are defined as tumor-associated macrophages (TAMs), which promote tumor formation. Li et al. found that Huaier can inhibit macrophage infiltration into the tumor microenvironment. Furthermore, Huaier could reduce the motility of macrophages by acting directly on them by measuring the expression of CD206, a marker of the M2 phenotype. In addition, Huaier can regulate macrophage polarization and increase phagocytosis of RAW264.7 cells (Li et al., 2015a). Another research also demonstrated that Huaier can upregulate inducible NO synthase (iNOS) activity in cholangiocarcinoma, significantly stimulating NO production by macrophages (Sun et al., 2013). The above studies indicate that Huaier has the ability to promote macrophage activation and inhibit their polarization toward the M2 phenotype.

### 4.2 Huaier regulates natural killer cells

Natural killer cells (NK cells) are an important component of intrinsic lymphocytes, which have MHC-independent killing activity, are antibody-independent, and recognize target cells non-specifically. The activation of NK cells during tumor development occurs through both direct recognition of malignantly transformed cancer cells as well as helper cells. (Tian, 2009). Using *in vitro* activity assays, Zheng et al. found that Huaier significantly increased NK cell activity (Zheng et al., 2014). In hepatocellular carcinoma, Huaier was able to significantly increase the number of NK cells and the effect was dose-dependent (Li et al., 2015a). Recently, NK cell-based tumor biotherapies have made considerable progress, and many new directions of tumor biotherapeutic pathways based on natural immune recognition of NK cells have been developed (Tian, 2009). The immunomodulatory properties exhibited by Huaier, based on influencing the activity and number of NK cells, further indicate its anti-tumor ability. Therefore, the role and mechanism of Huaier on NK cells in breast cancer needs to be further investigated.

### 4.3 Huaier regulates dendritic cells

Dendritic cells (DC cells) are specialized antigen-presenting cells (APCs) that activate initial T cells without specific cell surface molecular markers and are able to efficiently uptake, process, and deliver antigens. Immature DC cells have a strong migratory capacity, while Immune responses are initiated, regulated, and maintained by mature DC cells, which can effectively activate the initial T cells. Furthermore, DC cells can activate NK cells by acting as helper cells (Terme et al., 2008; Schwartzberg et al., 2009). A study by Pan et al. found a significant increase in the level of DC cell infiltration in the tumor microenvironment of 4T1 breast cancer mice in the Huaier treatment group. Furthermore, Huaier enhanced the level of co-stimulatory molecules expressed by DC cells, upregulated the expression of their surface markers which are signs of DC cell maturation (Banchereau and Steinman, 1998), and promoted pro-inflammatory cytokines such as IL-1β and IL-12p70 by DC cells, demonstrating that DC cells can mature *in vitro* with Huaier (Pan et al., 2020).

### 4.4 Huaier regulates lymphocytes

Adaptive immunity is an important regulator of the body against tumors and can be divided into humoral immunity and cellular immunity. Cellular immunity is mainly an immune response mediated by T lymphocytes, implying that T cells are stimulated by antigen, differentiate, proliferate, and transform into cytotoxic T cells, which directly kill antigen and exert a synergistic killing effect through the release of cytokines. CD4^+^ T lymphocytes are known as helper T lymphocytes (Th) becuase they play a key role in the initiation, final expression, and strength of the immune response by secreting cytokines and through the expression of surface molecules. Although CD8^+^ T lymphocytes can also secrete some Th-secreted cytokines, their most important function is to directly kill target antigens (e.g., viruses and tumor cells) and are therefore called cytotoxic T lymphocytes (CTL).

**FIGURE 6:**
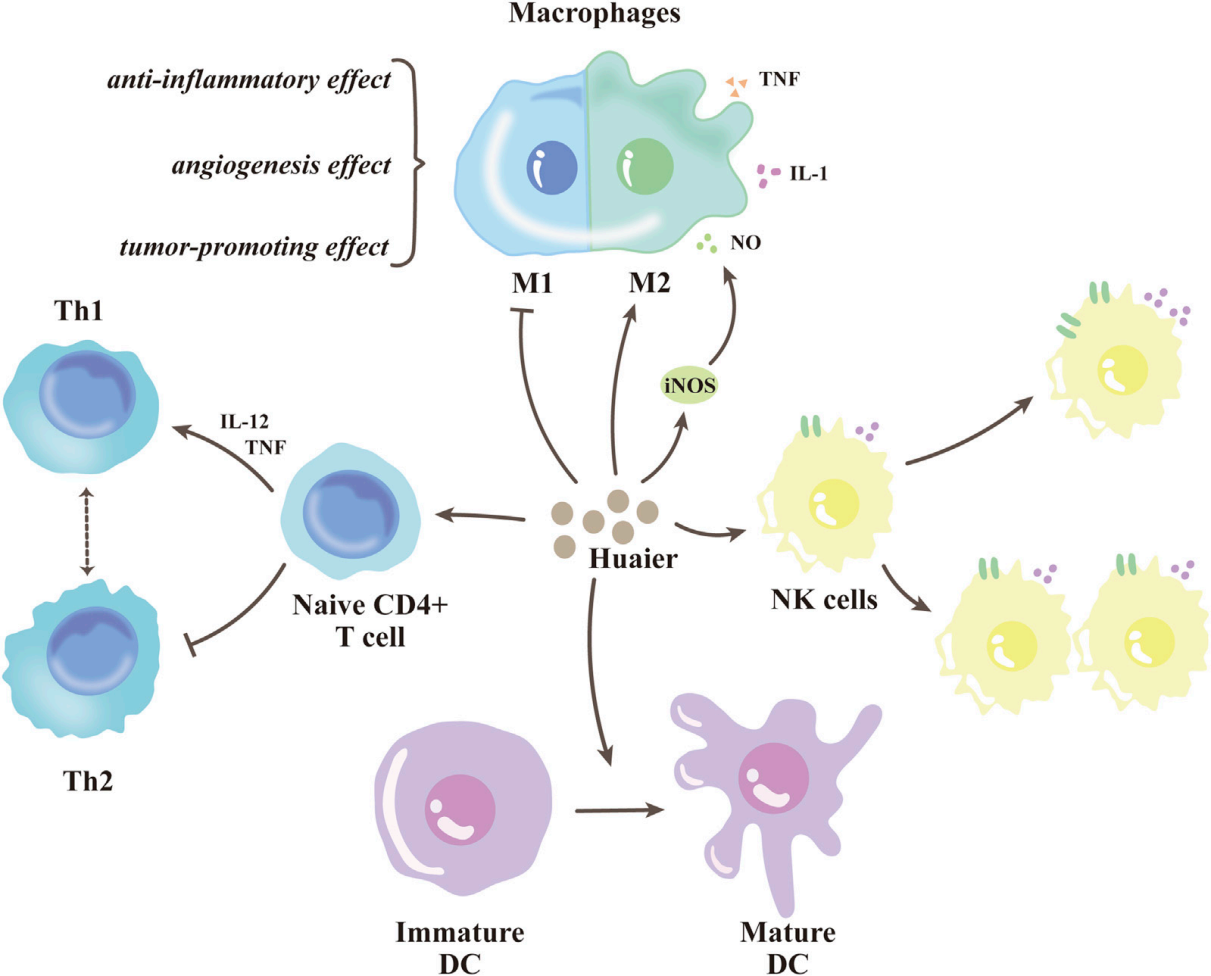
Huaier enhances anti-tumor immune response within breast cancer.

The reduced CD4+/CD8+ T lymphocyte ratio reported in clinical studies of patients with cancer suggests that these patients have suppressed cellular immune function and dysfunctional T cells, which make it difficult to clear abnormal cells and contribute to the development of cancer and its progression (Kong et al., 2005; Zhou and Xu, 2012). Following intraperitoneal injection of Huaier into H22 tumor-bearing mice, Li et al. showed that Huaier significantly increasing the number of CD4^+^ T cells while decreasing the number of CD8^+^ T cells. This change showed a dose-dependent effect on Huaier, suggesting that Huaier can increase the CD4+/CD8+ T cell ratio (Li et al., 2015a). Furthermore, existing studies have shown that Huaier can proliferate allozygous CD4^+^ T cells by affecting DC cells and induce the differentiation of naive CD4^+^ T cells towards the Th1 subpopulation, increasing the level of Th1 cells while suppressing the level of Th2 cells (You et al., 2009; Pan et al., 2020) (Figure 6).

## 5 The use of huaier in the clinical treatment of breast cancer

Recent studies have shown that Huaier not only exerts antitumor effects through several pathways, but also has the advantages of being safe, low toxic, and easy to administer, and has achieved good efficacy when widely used in clinical practice (Chen et al., 2022). Clinical studies have demonstrated the effectiveness of adjuvant treatment with Huaier in preventing HCC recurrence after radical hepatectomy (Zhang et al., 2021). Patients with hepatocellular carcinoma who receive Huaier can extend their recurrence-free survival and reduce their risk of extrahepatic recurrence (Zhang et al., 2021; Li et al., 2022; Luo and Hu, 2023). In addition, adjuvant therapy with Huaier can significantly improve immune function as well as conventional anticancer therapies’ efficacy and safety (Pu et al., 2022). In the treatment of breast cancer, conventional treatment combined with Huaier significantly improved overall remission and the health-related quality of life for patients compared to that obtained via conventional treatment alone (Yao et al., 2020), and Huaier adjuvant therapy has also been shown to improve immunity and enhance sensitivity to radiotherapy (Zhang et al., 2019; Li et al., 2020) (Table 1).

**TABLE 1:** Included clinical studies on the use of Huaier in breast cancer treatment.

Included literature	Sample size (T/C)	Intervention		Intervention time	Closing indicators
		T	C		
Wang et al. (2019a)	101/100	Post-operative chemotherapy + Huaier Granules	Post-operative chemotherapy	6/18 months	①②
Zhang et al. (2019b)	140/144	Chemotherapy + Huaier Granules	Chemotherapy	6 months	①⑫
Zhao and Yao (2020)	31/31	Pyrotinib + PCb Solutions + Huaier Granules	Pyrotinib + PCb Solutions	15 weeks	①⑪⑮⑬
Yang (2017)	30/30	Post-operative TP Solutions + Huaier Granules	Post-operative TP Solutions	6 weeks	⑧
Chen et al. (2004)	16/22	CTF Solutions Neoadjuvant chemotherapy + Huaier Granules	Neoadjuvant CTF Solutions	15 weeks	⑧
Dai and Cun (2007)	34/34	Post-operative VE Solutions + Huaier Granules	Post-operative VE Solutions	3 months	⑪
Xu and Tang (2009)	32/28	TAC Solutions Neoadjuvant chemotherapy + Huaier Granules	TAC Solutions Neoadjuvant chemotherapy	24 weeks	⑪⑬
Fu et al. (2005)	43	Chemotherapy + Huaier Granules	Chemotherapy	12 weeks	⑪
Liang et al. (2015)	49/49	Chemotherapy + Huaier Granules	Chemotherapy	6 Months	①⑪
Tang et al. (2006)	25/25	Operation + Huaier Granules	Operation	7 weeks	⑪
Shan et al. (2018), Changyou et al. (2018)	46/46	Neoadjuvant chemotherapy + Huaier Granules	Neoadjuvant chemotherapy	12 weeks	⑧⑭⑯
Yin et al. (2013)	20/20	Operation + Huaier Granules	Operation	Unknown	⑤⑨⑩
Zhang et al. (2014)	32/32	Post-operative chemotherapy + Huaier Granules	Post-operative chemotherapy	12 weeks	②⑤⑭
Chen and Liu (2020)	50/50	AP Solutions + Huaier Granules	AP Solutions	6 weeks	①⑥⑦⑫⑭
Li and Wang (2022)	42/42	AP Solutions + Huaier Granules	AP Solutions	6 weeks	②⑫⑬⑭
Guan et al. (2011)	88/77	Post-operative chemotherapy + Huaier Granules	Post-operative chemotherapy	3–6 months	①②③④⑤
Li et al. (2021b)	40/40	Post-operative Table Zoopi Star + Paclitaxel + Huaier Granules	Post-operative Table Zoopi Star + Paclitaxel	6 months	①②⑱
Wang et al. (2019b)	48/48	Post-operative FEC Solutions + Huaier Granules	FEC Solutions	6 months	①②⑥⑦⑫⑭⑰
Zhou et al. (2012)	39/40	Post-operative Letrozole + Huaier Granules	Post-operative Letrozole	6 months	⑤
Tian et al. (2015)	40/40	Post-operative Paclitaxel + Pirarubicin + Huaier Granules	Paclitaxel + Pirarubicin	Unknown	⑤⑭

Note: T = experimental group; C = Control group. PCb, Paclitaxel + Kaplan; TP, Paclitaxel + Kaplan; CTF, Cyclophosphamide + Pyrrolizidine + Fluorouracil; VE, Gemcitabine + V-ADM; TAC, Docetaxel + Pyrrolizidine + Cyclophosphamide; AP, Pemetrexed + Cisplatin; FEC, Fluorouracil + Table Zoopi Star + Cyclophosphamide.

①DFS. ②OS. ③RFS. ④DMFS. ⑤Recurrence and metastasis rate. ⑥CR. ⑦PR. ⑧ORR. ⑨Healing rate. ⑩Disease and death rate. ⑪Immune function. ⑫Tumor Markers. ⑬Quality of life. ⑭Toxic side effects. ⑮Drug-resistant proteins. ⑯MMP-2, MMP-9, in serum. ⑰VEGF. ⑱Endocrine hormones.

### 5.1 Huaier in combination with chemotherapy

The concept of “network pharmacology” has been proposed in the field of cancer treatment. This concept focuses on combination therapy marking a shift from “single-target drug” to “multi-target group therapy,” and Chinese medicine has been promoted as a supplement and alternative to anti-tumor therapy. (Qi et al., 2015; Wang et al., 2018; Zhang et al., 2019). Various tumors can be effectively treated with Huaier as an adjuvant (Chen et al., 2018), particularly in breast cancer clinical treatment (Tanaka et al., 2017; Wang et al., 2019; Yao et al., 2020).

Many trials have demonstrated that the combination of Huaier increases the efficacy of chemotherapy (Guan et al., 2011) or neoadjuvant chemotherapy (Chen et al., 2018; Zhang et al., 2019) and has a higher safety profile compared to that with chemotherapy regimens alone. In our previous clinical trial, chemotherapy in combination with Huaier was found to be effective in improving DFS and OS in patients with intermediate to advanced chemotherapy. Furthermore, the prolonged duration of dosing was effective in reducing the likelihood of disease progression, implying that Huaier has the potential to improve postoperative life quality and prognosis for the future in patients with intermediate to advanced TNBC(Wang et al., 2019). Huaier combined with AP regimen (pemetrexed disodium and cisplatin) can significantly reduce tumor marker levels and improve response rate and patient survival with breast cancer (Chen and Liu, 2020; Li and Wang, 2022). A clinical trial demonstrated that Huaier treatment combined with chemotherapy can effectively reduce serum tumor marker levels in those suffering from breast cancer and reduce the rate of recurrence and metastasis and prolong disease-free survival (Zhang et al., 2019). Clinical trials have also shown that Huaier can improve the adverse effects of chemotherapy, reduce the expression levels of CA199, CEA, and VEGF, reduce the adverse effects of chemotherapy, improve the immune function of patients (Li C. et al., 2020), and improving the patient’s physical and emotional state (Zhang et al., 2019).

The most common treatment for metastatic breast cancer is paclitaxel (Willson et al., 2019). Liu et al. found that Huaier increased the efficacy of paclitaxel in MCF-7 and MDA-MB-231 cell lines (Yang et al., 2017). Paclitaxel induces cell cycle arrest in the G2/M phase, whereas Huaier inhibits the cell cycle in the G0/G1 phase. Thus, these two drugs can have synergistic effects and inhibit tumor proliferation. c-Met is a signaling pathway that is frequently activated in tumor cells and can contribute to tumor formation, aggressive growth, and metastasis. p65 (RelA) on the NF-κB signaling pathway is a common signaling molecule with oncogenic properties. As the study progressed, researchers found that paclitaxel significantly reduced c-Met protein levels and IκBα levels, while p65 was significantly increased. However, Huaier suppressed the downregulation of IκBα, decreased the expression of p65, and enhanced the inhibitory effect on c-Met (Yang et al., 2017). Similar results were observed by Chen et al. in animal experiments, demonstrating that Huaier significantly inhibited the uptake of glucose by breast cancer cells when used in combination with paclitaxel. The authors also suggested that Huaier could reverse paclitaxel-like drug resistance through the PI3K/AKT pathway (Chen et al., 2018). Experimental results also showed that doxorubicin (Li et al., 2009) and pyrrolizidine (Zhao and Yao, 2020) resistance could be reversed by Huaier, confirming that Huaier is an effective chemotherapy resistance reversal agent. Patients treated with EP (epirubicin and paclitaxel) chemotherapy regimen after breast cancer surgery in combination with the combination of Huaier granules show better endocrine hormone recovery than those undergoing chemotherapy alone, as well as significantly improved survival time, disease-free progression survival, and 1-year survival rate (Li et al., 2009). A clinical trial by Yang et al. also confirmed that compared to the control group based on a TP regimen alone (paclitaxel, carboplatin), the experimental group combination of treated with Huaier had a lower incidence of adverse effects, less hematologic toxicity, and higher KPS scores (Yang, 2017).

Combining Huaier with 5-fluorouracil to treat cholangiocarcinoma can inhibit the expression levels of N-cadherin, vimentin, MMP-2, and MMP-9 through the STAT3 pathway, thereby suppressing tumor metastasis (Fu et al., 2019). Clinical trials have also demonstrated the efficacy of fluorouracil in the postoperative adjuvant treatment of metastatic breast cancer (Mafi et al., 2023). The CTF regimen (cyclophosphamide, pirarubicin, and fluorouracil) is one of the commonly used regimens for breast chemotherapy, and the results of clinical studies have shown an overall remission rate of 90.9% for Huaier combined with a CTF regimen for breast cancer, which is a significant improvement over the chemotherapeutic effect of the CTF regimen alone (68.8%) was significantly improved (Chen et al., 2004).

The immune function is generally depressed after chemotherapy for breast cancer, and clinical trials have demonstrated that Huaier polysaccharide can effectively improve immune function in these patients. Compared to the postoperative 3 cycles of VE chemotherapy regimen alone (gemcitabine 30 mg/m2 iv d1, 8+E-ADM 60 mg/m2 iv d2), patients who also underwent oral treatment with Huaier had significantly higher CD4^+^ levels, CD4+/CD8+ ratio, and NK cells in peripheral blood, with significant differences (Dai and Cun, 2007). The same results were observed when combining Huaier with TAC neoadjuvant therapy (Xu and Tang, 2009). Huaier also resulted in a significant increase in T-cell esterase and helper T cells in patients undergoing chemotherapy and also demonstrated good efficacy in treating the decrease in leukocyte and hemoglobin levels caused by myelosuppression and in reducing the side effects of chemotherapy (Fu et al., 2005; Liang et al., 2015). Furthermore, a study found that patients recovered their immune function more rapidly after treatment with Huaier (Tang et al., 2006).

The combination of neoadjuvant chemotherapy (pirenzosin + paclitaxel intravenously) with Huaier pellets is expected to improve the risk of recurrence and metastasis after chemotherapy for breast cancer (Li et al., 2009). As compared to the control group, MMP-2 and MMP-9 levels in patients’ serum were significantly lower as well, suggesting a more significant near-term and long-term efficacy and a higher safety profile of Huaier granules in combination with neoadjuvant chemotherapy for breast cancer patients (Shan et al., 2018).

Our analysis of the available studies on Huaier concluded that a variety of components in Huaier have strong antioxidant activity, which can reduce the oxidative stress caused by chemotherapeutic drugs, reducing chemotherapy-related side effects. Huaier can also regulate the immune function of the body, enhance the immunity of the body, and reduce the damage to the immune system caused by chemotherapy drugs, thus mitigating the side effects of chemotherapy. Furthermore, the polysaccharides in Huaier have an anti-inflammatory effect, which can reduce the inflammatory reaction caused by chemotherapy drugs, thereby reducing symptoms of chemotherapy. Some components in Huaier can protect the liver from the toxic damage of chemotherapy drugs, thus reducing chemotherapy symptoms. Therefore, Huaier, as a proprietary Chinese medicine, can not only enhance the efficacy of chemotherapy drugs, but also effectively reduce the side effects of conventional chemotherapy drugs.

### 5.2 Huaier in combination with radiotherapy

Currently, radiotherapy plays an important role in the systemic treatment of breast cancer and is commonly used to facilitate surgical resection of tumors prior to surgery and to control residual microscopic malignancies after surgery. During breast cancer treatment, radiotherapy has been shown to reduce local recurrence rates; however, radiotherapy also causes reactive oxygen-dependent damage to DNA and other molecules, which may eventually lead to permanent inactivation of cell division or even initiation of cell death programs, and therefore, its radiotoxic nature limits widespread use (O'Donovan et al., 2017). A study by Sun et al. compared 3-year disease-free survival, overall survival, and recurrence and metastasis rates of patients with breast cancer undergoing radiotherapy treated with adjuvant therapy with and without Huaier. The results indicated that Huaier particles could prolong disease-free survival and reduce recurrence and metastasis rates in radiotherapy patients (Sun, 2011). Using HTA2.0 transcriptome microarray analysis, Ding et al. found that as a result of Huaier’s actions, the expression of genes associated with cell cycle, cell division, and cell cycle phases could be downregulated. Following radiotherapy, using Huaier significantly prolonged the duration of its γ-H2Ax lesions and disrupted homologous recombination (Ding et al., 2016). Therefore, Huaier may be a potential radiosensitizer for the treatment of patients with breast cancer. Available clinical trials have also demonstrated that the combination of Huaier after radiotherapy significantly improves the outcome of breast cancer in older patients and reduces recurrence and metastasis rates as well as treatment side effects (Yin et al., 2013; Zhang et al., 2014).

Radiation-treated cancer cells release large amounts of antigenic substances, which are immunostimulatory signals that support tumor-targeted immune responses (Wennerberg et al., 2017). A growing number of clinical studies suggest that the ultimate efficacy of radiotherapy may depend on the immune system of the patient. Survival of cancer cells after radiotherapy depends on the ability to respond to the damage caused by radiotherapy by avoiding cellular senescence or by modulating cytoprotective pathways (Petroni et al., 2022). Huaier is an effective immunomodulator (Li et al., 2022), the evidence to date suggests that Huaier will be effective in enhancing the efficacy of radiotherapy and improving the long-term prognosis of patients after radiotherapy. Further clinical trials are required to corroborate this.

### 5.3 Huaier in combination with endocrine therapy

Treatments that target the hormone receptors in breast cancer cells are among the main therapies for early-stage breast cancer. Inhibitors of estrogen receptors, such as tamoxifen, inhibit the growth and spread of breast cancer cells (Viedma-Rodríguez et al., 2014), and androgen receptor antagonists such as anastrozole can inhibit the stimulation of breast cancer cells by androgens (Nabholtz, 2006). In recent years, it has been shown that Huaier combined with endocrine therapy can significantly improve the outcome of breast cancer patients with positive ER and/or PR. There is evidence from existing clinical trials that letrozole combined with Huaier granules treatment can effectively improve the 3-year recurrence and metastasis rate of breast cancer patients (Zhou et al., 2012). Tamoxifen, a widely used anti-estrogen drug, is associated with toxicity, adverse effects, and drug resistance (Piva et al., 2014). Breast cancer drug resistance may be related to ER coactivator dysregulation and loss of ER expression (Gutierrez et al., 2005; Osborne and Schiff, 2011). New evidence suggests that PI3K/AKT/mTOR and ER signaling pathways intersect at multiple junctions and exhibit a high degree of interdependence (Simoncini et al., 2000; Campbell et al., 2001). Qi et al. demonstrated through *ex vivo* experiments that tamoxifen used in combination with Huaier for breast cancer treatment significantly increased inhibition of AKT/mTOR signaling by Huaier, enhancing the effect of tamoxifen (Qi et al., 2016). Gao et al. showed that Huaier can increase ataxia capillaris mutation by inhibiting miR-7, thereby inhibiting the proliferation of endocrine drug-resistant cells (Gao et al., 2017). Therefore, the experimental results demonstrated that Huaier can improve the resistance of breast cancer cells to endocrine therapeutic agents. In addition, the combination of Huaier and endocrine therapy not only enhances the therapeutic effect, but also effectively alleviates the side effects of endocrine drugs: Huaier granules have the effect of clearing heat, detoxifying, cooling blood, and dispersing nodules, which can alleviate the side effects of endocrine drugs, particularly symptoms such as hot flushes (Qi et al., 2016).

It should be noted that studies on the combination of Huaier with endocrine therapy are relatively limited, and the efficacy and safety need to be verified through further clinical studies.

### 5.4 Huaier in combination with targeted therapy

Following recent developments in research on the molecular biology of signaling pathways and apoptosis in tumor research, anti-breast cancer research is focusing on molecular targets and targeted therapies. Polutinib is a tyrosine kinase inhibitor (TKI)-based targeted agent recommended for the second-line treatment of advanced HER2-positive breast cancer (Yan et al., 2022). Clinical studies have confirmed that pyrotinib-targeted therapy for breast cancer combined with Huaier granules significantly improves immune function and reduces the expression level of drug-resistant genes, improving patient quality of life (Zhao and Yao, 2020). Everolimus, the first oral targeted therapy drug available for advanced HR+/HER2-breast cancer is everolimus, is an inhibitor of the target of rapamycin (mTOR) protein (Ma et al., 2023), and current clinical trials have demonstrated its efficacy in breast cancer-based endocrine therapy (François-Martin et al., 2023). Another commonly used agent is alpelisib, a specific PI3K inhibitor (Bello Roufai et al., 2023; Cerma et al., 2023). HR + breast cancers are more likely to have mutations in PI3K/AKT/mTOR than other isoforms (Cerma et al., 2023). Wang et al. confirmed that Huaier can lead to autophagic cell death in breast cancer cells by inhibiting the mTOR downstream targets (Wang et al., 2015). Hu et al. also showed that Huaier suppressed the tumorigenic capacity of activated mTOR cells *in vivo* (Hu et al., 2016). Furthermore, breast cancer cells are more sensitive to chemotherapeutic agents when Huaier activates mTOR signaling, since tamoxifen also induces autophagy and apoptosis in ER-positive breast cancer cells via the AKT/mTOR signalling pathway (Nunnery and Mayer, 2020). Therefore, the evidence to date promotes the synergistic antitumor effects of Huaier in breast cancer therapy. However, further research is needed to corroborate this hypothesis.

## 6 Conclusion and prospects

In this review, we systematically summarized the anti-tumor mechanisms of Huaier, demonstrate its potentials in the field of anti-breast cancer treatment. In order for Huaier to be widely used in clinical trials, some issues must be addressed. First, the sample size of the available clinical trials of huaier are small. Furthermore, studies on the use of Huaier in breast cancer treatment were mainly conducted in vitro cell models or animal models and there are limited non-experimental studies; hence, the level of evidence is low. Therefore, further large clinical trials are required to verify the effectiveness of Huaier in humans in order to further promote its clinical use. Secondly, there is a lack of research on underlying mechanisms of the Huaier in breast cancer treatment, and further research needs to investigate its role in The process of regulating tumor cell proliferation, apoptosis, invasion, and metastasis. Robust evidence is needed to provide a more reliable theoretical basis for clinical treatment. There is also a lack of clinical guidelines and standardized use protocols, and it is important to develop guidance to ensure safety and efficacy in the treatment process.

Based on the evidence on the use of Huaier to date, we support the following directions of research in the field of breast cancer: (1) investigation of the chemical structure and active components of Huaier to determine its inhibitory effect on breast cancer cell; (2) exploration of the molecular mechanism of Huaier in breast cancer treatment through *in vivo* and *in vitro* experiments and comparisons with existing breast cancer treatment; (3) combining Huaier with existing treatment approaches, such as chemotherapy, radiotherapy, endocrine therapy and immunotherapy, to investigate its synergistic effects breast cancer treatment; (4) large-scale clinical trials to confirm the effectiveness of Huaier in the treatment of breast cancer. Focusing on these areas of research will contribute to the development of more effective clinical treatment regimens for breast cancer treatment using Huaier polysaccharides and promote the clinical translational application of Huaier polysaccharides.
